# Differential Effects of D-Cycloserine and ACBC at NMDA Receptors in the Rat Entorhinal Cortex Are Related to Efficacy at the Co-Agonist Binding Site

**DOI:** 10.1371/journal.pone.0133548

**Published:** 2015-07-20

**Authors:** Alex M. Lench, Emma Robson, Roland S. G. Jones

**Affiliations:** Department of Pharmacy and Pharmacology, University of Bath, Bath, United Kingdom; Creighton University, UNITED STATES

## Abstract

Partial agonists at the NMDA receptor co-agonist binding site may have potential therapeutic efficacy in a number of cognitive and neurological conditions. The entorhinal cortex is a key brain area in spatial memory and cognitive processing. At synapses in the entorhinal cortex, NMDA receptors not only mediate postsynaptic excitation but are expressed in presynaptic terminals where they tonically facilitate glutamate release. In a previous study we showed that the co-agonist binding site of the presynaptic NMDA receptor is endogenously and tonically activated by D-serine released from astrocytes. In this study we determined the effects of two co-agonist site partial agonists on both presynaptic and postsynaptic NMDA receptors in layer II of the entorhinal cortex. The high efficacy partial agonist, D-cycloserine, decreased the decay time of postsynaptic NMDA receptor mediated currents evoked by electrical stimulation, but had no effect on amplitude or other kinetic parameters. In contrast, a lower efficacy partial agonist, 1-aminocyclobutane-1-carboxylic acid, decreased decay time to a greater extent than D-cycloserine, and also reduced the peak amplitude of the evoked NMDA receptor mediated postsynaptic responses. Presynaptic NMDA receptors, (monitored indirectly by effects on the frequency of AMPA receptor mediated spontaneous excitatory currents) were unaffected by D-cycloserine, but were reduced in effectiveness by 1-aminocyclobutane-1-carboxylic acid. We discuss these results in the context of the effect of endogenous regulation of the NMDA receptor co-agonist site on receptor gating and the potential therapeutic implications for cognitive disorders.

## Introduction

The entorhinal cortex (EC) acts as a crucial dynamic processer of information entering and leaving the hippocampus, controlling its interaction with the rest of the neuraxis. This intimate association means that the EC plays a pivotal role in declarative and spatial memory, particularly spatial cognition, representation and navigation, and in other cognitive processes such as attention, and conditioning [1–5]. Dysfunction of the EC and of EC-hippocampal interactions has been implicated in many pathological conditions, particularly initiation and propagation of temporal lobe seizures and the cognitive abnormalities/decline associated with a variety of psychiatric/neurological disorders (including schizophrenia, bipolar and depressive disorders, Alzheimer’s disease, Parkinson’s disease, Huntington’s disease, amyotrophic lateral sclerosis etc.; see refs in [6]). This laboratory has a longstanding interest in control of neuronal activity and excitability in entorhinal networks. One focus of this research has been the role and regulation of NMDA receptors (NMDArs) in both normal synaptic function and epileptic activity [6–8].

The NMDAr consists of two obligatory GluN1 subunits and two GluN2 subunits. A glutamate binding site is present on the GluN2 subunit whilst a co-agonist site that can be activated endogenously by both glycine and D-serine is located on the GluN1 subunit. There has been an interest in targeting the NMDAr co-agonist binding site for therapeutic treatment of CNS disorders (see [9]). Cognitive decline is a pronounced characteristic of Alzheimer’s disease and other dementias, but is also a feature of Parkinson’s disease, Huntington’s disease, stroke, amyotrophic lateral sclerosis, epilepsy, and psychosis [10–15]. D-cycloserine (DCS), which is used therapeutically as an antimicrobial agent, is a partial agonist at the NMDAr co-agonist binding site, and has been demonstrated to induce cognitive enhancement at low doses in behavioural and psychological studies [16–25]. Thus, it could be useful in treatment of neurodegenerative disorders involving cognitive decline, in therapy of mood and thought disorders such as schizophrenia and depression, and for augmenting psychotherapies for the treatment of drug addiction and anxiety disorders. On the other hand, DCS has also been suggested to have direct antiepileptic actions or be a useful adjunct in the treatment of epilepsy [26–28]. In this context it is interesting that epilepsy is often associated with cognitive impairment, and this may be related to a loss of D-serine and reduced co-agonist regulation of the NMDAr [29]. Thus, DCS could have a dual effect to reduce seizures and restore cognitive activity in people with epilepsy.

We have previously shown that presynaptic NMDArs (preNMDArs) on excitatory terminals can tonically facilitate the release of glutamate at EC synapses [30–32]. These receptors are predominantly diheteromeric GluN1-N2B receptors [8,31,32] whereas those located postsynaptically at the same sites may be largely triheteromeric GluN1-N2A-N2B receptors [8]. Recently, we have investigated the co-agonist regulation of the preNMDAr and shown that endogenous D-serine, released from astrocytes, is the preferred ligand for the co-agonist-binding site, which appears to be fully saturated by the ligand under baseline conditions [33]. Given the potential therapeutic relevance of the co-agonist binding site, we have now examined the effects of partial agonists at this site. We compared effects on the presynaptic NMDAr-mediated regulation of glutamate release in the EC to actions on NMDAr-mediated responses at postsynaptic sites (postNMDAr). Our data indicate a differential modulation of the receptors by the two partial agonists, which was apparently dependent on efficacy. These effects could be relevant to putative therapeutic actions of the ligands.

## Materials and Methods

### Ethics Statement

Experiments conformed with the U.K. Animals (Scientific Procedures) Act 1986, European Communities Council Directive 1986 (86/609/EEC) and were subject to conformity with the University of Bath ethical review document. The research reported in this study is not directly regulated by Home Office procedures, as it was all conducted using Schedule 1 procedures. Nevertheless, all experiments were subject to internal ethical review and were specifically approved by the University’s Animal Welfare and Ethical Review Body Committee. This ensures that the minimum number of animals is used and that every precaution is taken to reduce suffering and stress. All research involving use of animal tissue at the University of Bath requires a consideration of ethical implications by the principal investigator. A second investigator, external to the research group, reviews this and signatory approval from both the external reviewer and Head of Department, is required preceding further review by the Departmental Research Ethics Officer. The Ethics Officer submits a report to the Animal Welfare and Ethical Review Body Committee discussion with the investigator. These processes ensure that ethical implications of the research have been adequately considered and that there is a process in place for managing ethical issues. The Animal Users Committee monitors all licenced and Schedule 1 procedures at quarterly meetings.

### Slice preparation

Combined hippocampal-entorhinal brain slices from male or female juvenile Wistar rats (50–100g; P28–38) were prepared as described previously [34]. Following cervical dislocation, rats were decapitated and the brain rapidly removed into oxygenated artificial cerebrospinal fluid (aCSF; see below for composition) at 4°C. Slices (300 μm thick) were cut (Campden Vibroslice or Model 7000smz microtomes) at 4°C and transferred to a storage chamber containing oxygenated aCSF maintained at room temperature. They were allowed to recover for 1 hour before being used for electrophysiological recording. The aCSF contained (in mM) NaCl (126), KCl (4), MgSO_4_ (1.25), NaH_2_PO_4_ (1.4), NaHCO_3_ (24), CaCl_2_ (2), D-glucose (10), ascorbic acid (0.57), sodium pyruvate (5) and creatinine monohydrate (5). Ketamine (4 μM), indomethacin (45 μM), aminoguanidine (25 μM) and Coomassie Brilliant Blue (250 nM) were included in the cutting solution, and both cutting and storage solutions also contained the antioxidants, n-acetyl-L-cysteine (2 μM) and uric acid (100 μM), to aid neuronal survival and viability. We have previously established that the use of these additives facilitates production of robust and viable slices, but does not affect the pharmacology of glutamate transmission. Of particular concern is the possibility that residual ketamine may compromise studies of NMDAr responses in the experiments. However, we have used ketamine as a neuroprotectant, both as an anaesthetic prior to decapitation and in cutting solutions for over 20 years, and before introducing it into routine use investigated this extensively, finding no evidence that this was an issue. In our current protocols, slices are kept in a ketamine-free storage chamber for a minimum of 1 hour (in reality most are stored for 2 hours plus) before transfer and then washed in the recording chamber for a further 30–40 minutes before recording. The storage chamber has a large-sink circulating volume of around 40 ml and the recording chamber is perfused at 2 ml/min. The very high reverse rate constant for ketamine means that its dissociation from the receptor channel is likely to be rapid and complete before actual recording commences. The ability to elicit large repeatable NMDAr responses in the tissue suggests that residual ketamine is unlikely to be an issue.

### Whole-cell patch clamp recordings

After recovery, individual slices were transferred to a chamber on an Olympus BX50WI microscope and perfused (~2 ml/min) with oxygenated aCSF at 31–32°C, where they were allowed to further equilibrate before recording began. Neurones were visualised using differential interference contrast optics and an infrared video camera.

Whole cell patch clamp recordings were made with an Axopatch 200A or 200B amplifier using borosilicate glass pipettes pulled on a Flaming-Brown microelectrode puller. To record AMPA receptor (AMPAr) mediated miniature excitatory postsynaptic currents (mEPSCs) we used a Cs-gluconate based intracellular solution containing (in mM) D-gluconate (100), HEPES (40), QX-314 (1), EGTA (0.6), NaCl (2), Mg-gluconate (5), tetraethylammonium-Cl (5), phosphocreatinine (10), ATP-Na (4), GTP-Na (0.3) and MK801 (5mM). The solution was adjusted to pH 7.3 with CsOH and to 275–290 mOsm by dilution. Recordings were conducted in the presence of TTX (1 μM) to eliminate activity-dependent glutamate release. To permit recording of AMPAr-mediated responses in isolation, postNMDArs were blocked by inclusion of MK-801 in the patch pipettes. This also allowed us to monitor activity at preNMDArs without the complication of postsynaptic receptor activation/blockade. This approach was developed and validated in this laboratory [7,30–32], and has been used successfully by others [35–40].

Miniature EPSCs were recorded at a holding potential of -60mV, filtered at 2 kHz, digitised at 50kHz, and stored using AxoScope software. Access resistance was monitored at 5-minute intervals throughout recording and if it varied by greater than 15% neurones were excluded from analysis. Series resistance compensation was not used. Input resistances for the neurones recorded in these studies were of the order 500–700 MΩ. Data recording commenced 15–20 minutes after gaining whole cell access and then continued for at least 15 minutes during control and each drug treatment condition. mEPSCs were analysed offline using a threshold-crossing algorithm in Minianalysis software (Synaptosoft, Decatur) over stable 5-minute periods of recording. Average frequency was compared before and after drug application. Amplitudes of spontaneous currents were determined as median values for each neurone as these better reflect the population distributions (normal distribution with a skew towards larger amplitude events) than arithmetical means. Median values were then averaged for comparative illustrative purposes. We have used this approach in previous studies [41]. The kinetics of mEPSCs were compared via arithmetical means. Statistical comparisons of drug effects on mean frequency and kinetics and mean median amplitudes within cells employed a two-tailed paired t-test.

Evoked postsynaptic responses were elicited by electrical stimulation using a bipolar tungsten electrode placed on the surface of the slice lateral to the recording site in the EC. Bipolar pulses (10–30 mA, 100 ms duration) were delivered at intervals of 20 seconds. Again, recordings were made using whole cell patch clamp but without intracellular MK801 and extracellular TTX. To isolate evoked NMDAr-mediated EPSCs (eNEPSCs), neurones were recorded at a holding potential of +40 mV in the presence of NBQX (10 μM), bicuculline (20 μM) and picrotoxin (50 μM). That NMDArs mediated the recorded responses was confirmed by abolition with 2-AP5. After gaining whole cell access, no stimulation was applied for at least 20 minutes to allow series resistance and cell activity to stabilise. Drugs were applied by addition to the perfusion aCSF, and each concentration was perfused for 10 minutes before increasing to the next to construct cumulative concentration-response curves. The amplitude, rise time and decay time of eNEPSCs were analysed off-line using Minianalysis software (Synaptosoft, Decatur). In analysis of various treatments on decay time, this was calculated as the time taken to reach 37% of peak amplitude.

To determine the effects of drug treatments on eNEPSCs, in each neurone the last five events in each treatment epoch were averaged and the amplitude, rise time, decay time were determined. The mean and standard error were then calculated for the group and are given for overall measurements for data sets. The statistical significance of differences between control and drug treatments was assessed using a two-tailed paired t-test. Normalised values were also calculated as percentage change from control and these were used to calculate average percentage changes.

### Materials

Salts used in preparation of aCSF were purchased from Fisher Scientific (UK). Indomethacin, aminoguanidine, n-acetyl-L-cysteine, uric acid, Coomassie Brilliant Blue, and agents used in the preparation of the patch pipette solution, apart from QX-314 (Tocris UK) and MK801, were purchased from Sigma (UK). Ketamine was supplied by Fort Dodge Animal Health Ltd (Southampton, UK). Drugs were stored as frozen, concentrated stock solutions in distilled water and applied by dilution in the aCSF perfusion immediately before use. The following drugs were supplied by Tocris UK or Ascent Scientific UK: DCS, ACBC (1-aminocyclobutane-1-carboxylic acid), DCKA (5,7-dichlorokynurenic acid), 2-AP5 (D,L-2-amino-5-phosphonopentanoic acid), MK801, D-serine, TTX, NBQX (6-nitro-7 sulphamoylbenzo[f]quinoxalone-2,3-dione disodium), bicuculline methiodide and picrotoxin.

## Results

Experiments were conducted on neurones in layer II of the medial EC. We did not attempt to specifically identify neurones by dye injection. The selected neurones had identifiable pyramidal or stellate cell morphology when viewed with DIC and are likely to be glutamatergic principal neurones.

### Effects of DCS at presynaptic NMDArs

PreNMDArs tonically facilitate the spontaneous release of glutamate at excitatory synapses in the EC [30,31]. In a recent study we showed that the co-agonist site of the receptor is endogenously bound by D-serine. Thus, exogenous application of the full agonist, had little effect on spontaneous release, but co-agonist site antagonists reduced it, suggesting a saturation of the receptor/effect by endogenous D-serine [33].

**Fig 1:**
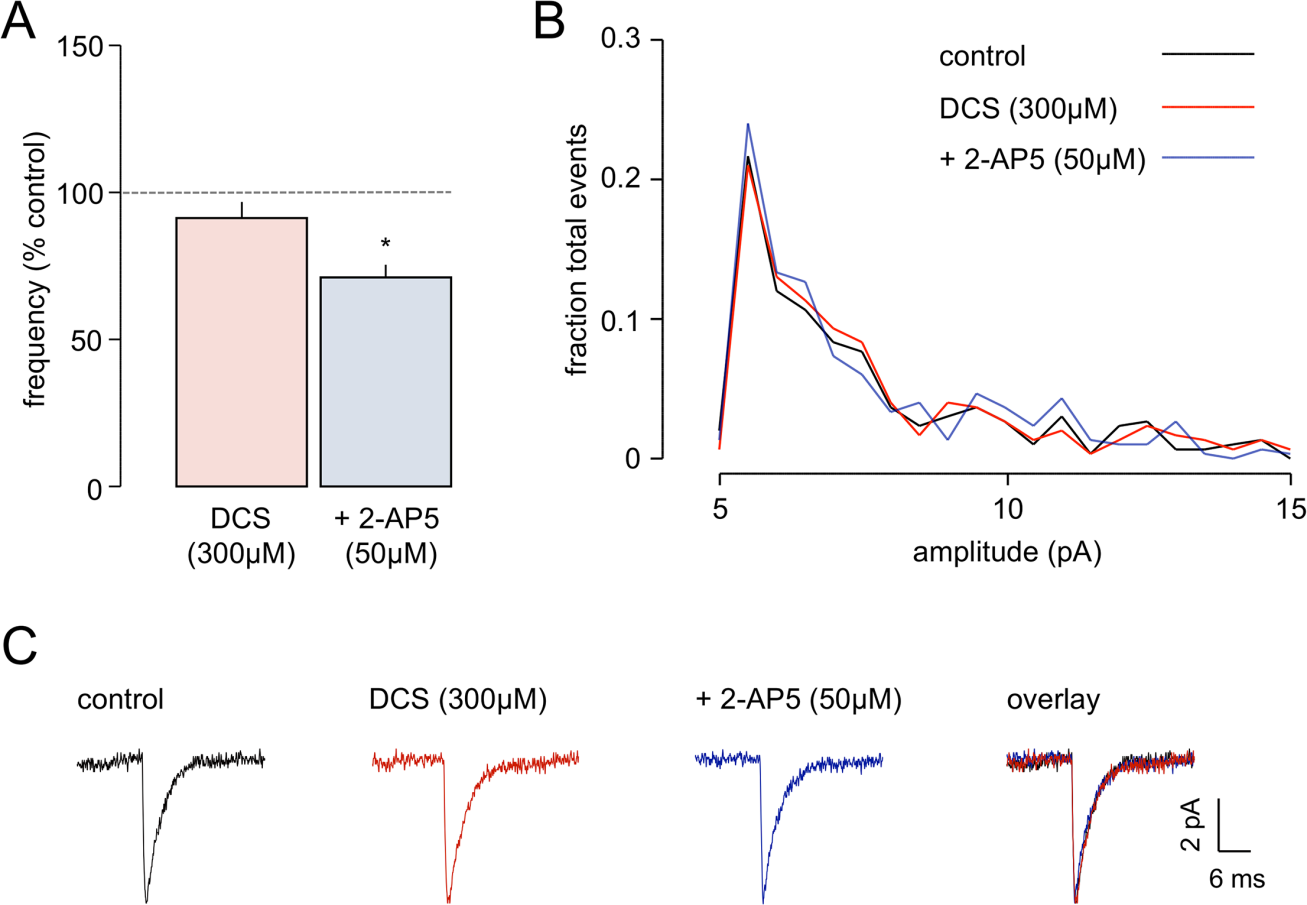
Effects of DCS on preNMDAr activity. **A.** The histograms show average normalised changes in frequency of mEPSCs with DCS and 2-AP5 in six neurones. DCS was without effect whereas subsequent, cumulative addition of 2-AP5 reduced frequency by ~30%. **B.** The graphs show that the frequency distribution of amplitudes of mEPSCs was unaltered by either antagonist. C. Average mEPSCs (n = 20) in one neurone showing the lack of change in amplitude, rise or decay time with either drug. * *P*<0.05.

In the first set of experiments we determined the effect of the partial agonist, DCS, using the frequency of mEPSCs mediated by AMPAr as a reporter for glutamate release and, hence, preNMDAr activity [30]. DCS (300 μM) had no observable effect on mEPSC frequency in 6 neurones (Fig 1A). In control conditions, mean frequency was 0.65±0.29 Hz and this was almost identical in the presence of DCS (0.62±0.32 Hz). However, subsequent addition of 2-AP5 (50 μM) reduced the frequency of mEPSCs to 0.47±0.23 Hz, a decrease to 71.1±4.3% of control (Fig 1A) confirming that preNMDArs were operative at these synapses to tonically facilitate glutamate release. DCS failed to alter either the mean amplitude of mEPSCs (7.0±0.4 pA *v* 6.9±0.4 pA), or their frequency distribution (Fig 1B and 1C), and these parameters were also unchanged with further addition of 2-AP5 (mean amplitude 6.9±0.4pA). Likewise, decay kinetics (control 2.22±0.32 ms) were unaltered, either by DCS alone (1.98±0.30 ms) or DCS plus 2-AP5 (2.00±0.28 ms). Finally, neither mEPSC rise time nor baseline holding current was altered in any situation (not shown). The lack of effect on kinetics is clearly shown by the averaged mEPSCs shown in Fig 1C. DCS was also tested, at a lower concentration (100 μM; n = 6) and again had no observable effect on mEPSC parameters (e.g. frequency 0.55±0.14 Hz in control and 0.53±0.19 Hz in DCS; other data not shown). These data confirm that glutamate release was subject to tonic facilitation by preNMDAr, but, at the concentrations tested, the partial agonist at the co-agonist binding site did not alter the effects of presynaptic receptor activation.

### Postsynaptic NMDAr mediated EPSCs in the EC

**Fig 2:**
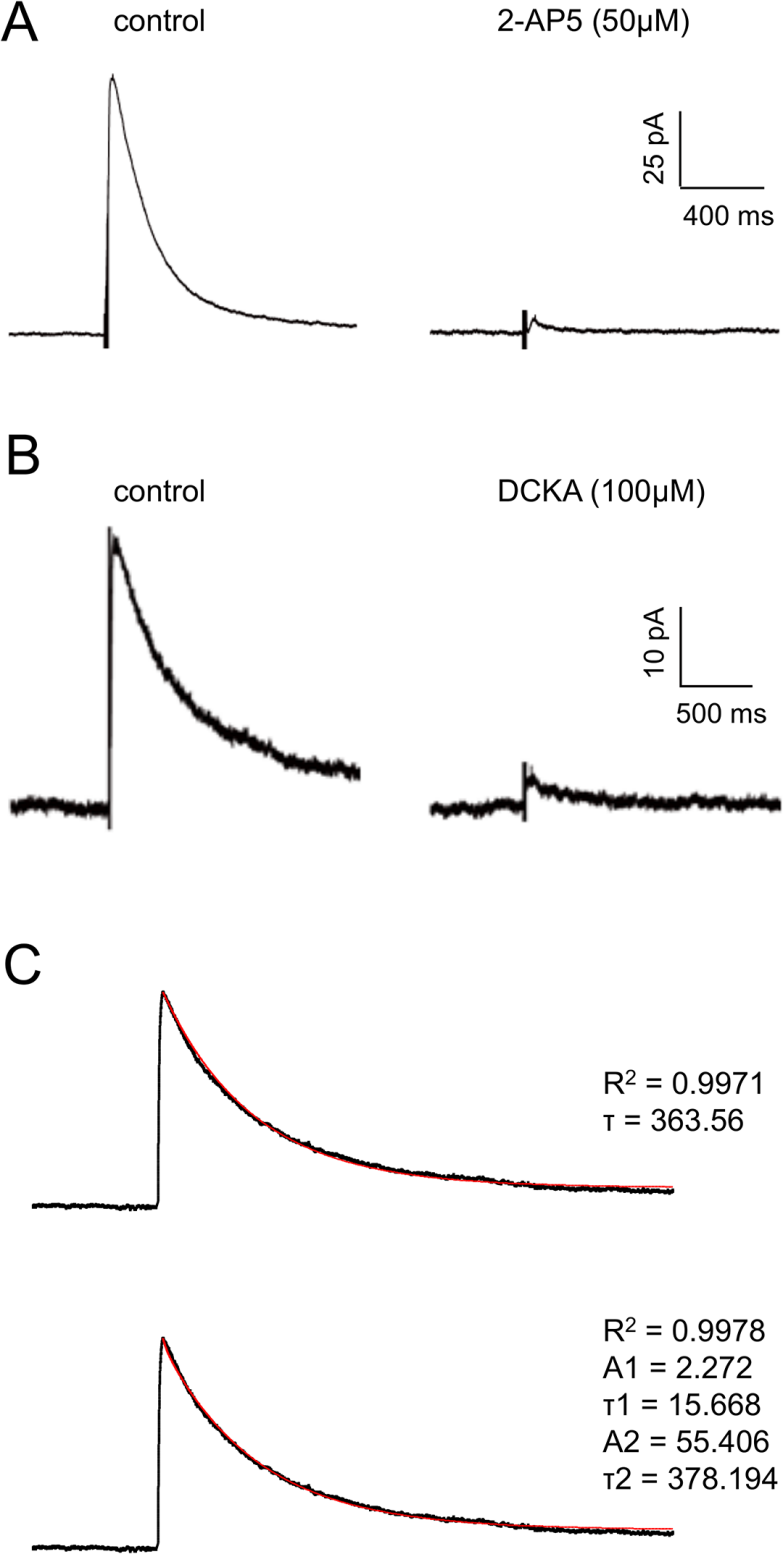
eNEPSCs in entorhinal neurones. The traces show voltage clamp recordings of eNEPSCs evoked in layer II neurones in the medial EC. Each response is the average of at least 5 events. **A.** The addition of the competitive antagonist, 2-AP5 abolished the eNEPSC. **B.** Likewise, application of the co-agonist site antagonist, DCKA, was also able to abolish the eNEPSC. **C**. The decay of n averaged (n = 8) eNEPSC was fitted with either a mono- (top) or bi-exponential function. Whilst R^2^ values show an excellent fit to a biexponential decay, the mono-exponential fit was almost as good. The slow decay corresponding to tau-2 in the biexponential fit was extremely variable from neurone to neurone, and precluded meaningful analysis. Since the mono-exponential fit was excellent, in all further studies we assumed a mono-exponential decay.

In the presence of AMPAr and GABA_A_ receptor blockers, stimulation in the EC lateral to the recording site elicited large, slowly decaying EPSCs in layer II neurones voltage clamped at +40 mV. These were abolished by 2-AP5, confirming that they were mediated by postNMDArs (Fig 2A; n = 3). In addition, we also tested the effect of a high concentration (100 μM, determined to be close to Emax) of the co-agonist site competitive antagonist, DCKA (Fig 2B; n = 3). This also rapidly (<5 minutes) abolished the eNEPSC and confirmed that the co-agonist site of the postNMDAr was endogenously activated. We have previously examined other aspects of the pharmacology of these eNEPSCs in entorhinal neurones and determined that they are likely to be mediated by triheteromeric GluN1-GluN2A-GluN2B receptors [8]. The slow decay of eNEPSC in other neurones has been shown to be bi-exponential [42] so we looked at this possibility in our neurones. We found that the decay of the eNEPSC in layer II was only marginally better described by biexponential decay kinetics, compared to monoexponential (Fig 2C). The decay time constants were variable from neurone to neurone, particularly in the case of the slow phase of decay, where the variability precluded any meaningful analysis, so in all studies we have assumed monoexponential decay, and compared the time taken to decline to 37% of peak amplitude.

### Effects of D-serine at postsynaptic NMDArs

**Fig 3:**
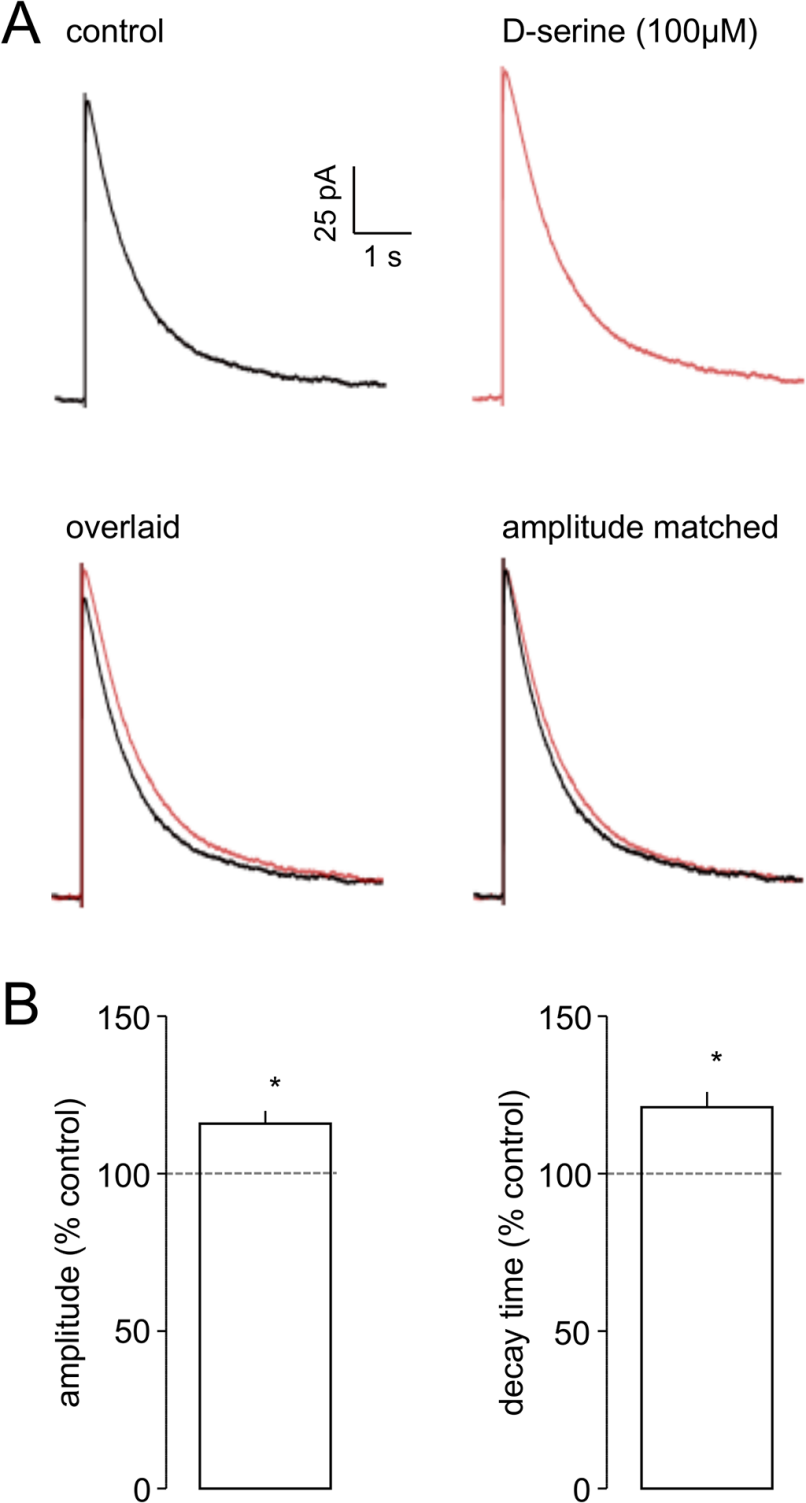
D-serine increases the amplitude and decay time of eNEPSC in EC neurones. **A.** The traces show averaged eNEPSCs recorded in one neurone. A small, but clear, increase in amplitude by D-serine can be seen when the responses are overlaid. When control and D-serine responses were scaled to the same amplitude and overlaid, the prolongation of decay time is also apparent. **B.** The histograms show pooled normalised data for eNEPSC amplitude and decay times in the presence of D-serine in six neurones. * *P*<0.05.

As noted above, we have previously shown that the co-agonist site of the preNMDAr is essentially saturated (or very close to it) by endogenous D-serine [33]. However this did not appear to be the case with the postNMDArs at EC synapses. Thus, exogenous application of the full agonist, D-serine (100 μM, n = 6), increased the mean peak amplitude of eNEPSCs, from 205.5±40.7 pA to 240.3±50.1 pA (*P*<0.05), a normalised increase of 15.9±4.0% (Fig 3). The averaged responses from one study are shown in Fig 3A, where an increase in peak amplitude is evident. In addition, D-serine prolonged the decay time of the eNEPSC from 381.3±105.0 ms to 450.5±112.3 ms (Fig 3B). Scaling the traces to match control and drug treated amplitudes shows this prolongation of decay. In contrast, the rise time of the eNEPSC in control recordings was 36.2±8.0 ms, and this was unaltered (33.4±6.3 ms) in the presence of D-serine.

### Effects of DCS at postsynaptic NMDArs

**Fig 4:**
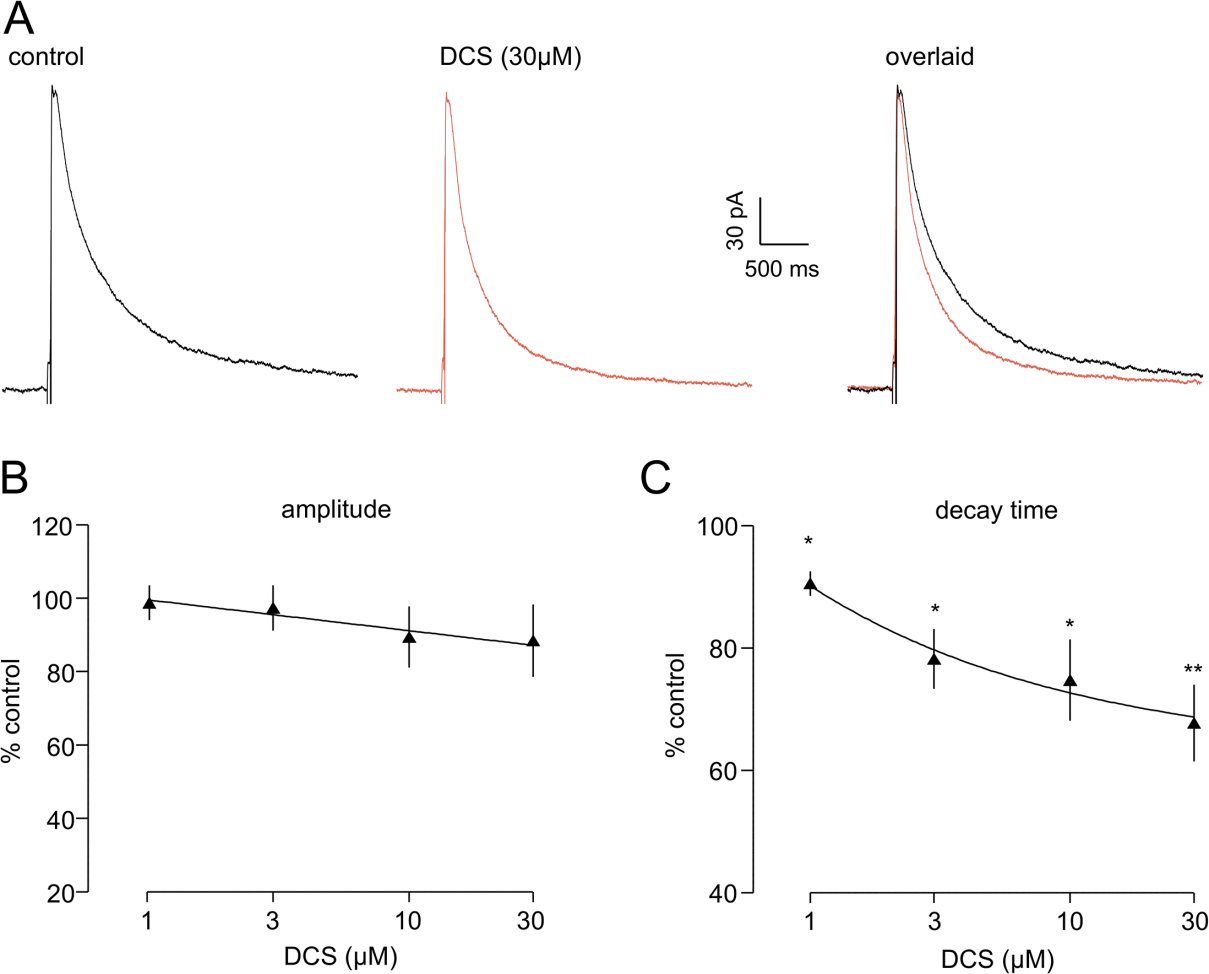
DCS decreases the decay time of eNEPSCs. **A.** Averaged traces for one neurone are shown for control and in the presence of 30 μM DCS. The overlaid traces clearly show that the amplitude of the eNEPSC is unaffected whereas the response decay is substantially accelerated. The graphs show pooled data of cumulative concentration response curves for normalised data in six neurones for peak amplitude **(B)** and decay time **(C)**. DCS failed to significantly alter peak amplitude at any concentration, but produced a concentration-dependent decrease in eNEPSC decay time. * *P*<0.05; ** *P*<0.01.

We next examined the effect of DCS (1–30 μM), a partial agonist at the co-agonist site, on eNEPSCs in 6 neurones. In contrast to D-serine, DCS did not significantly alter the amplitude of eNEPSCs at any concentration tested, with a small reduction of around 8% recorded at the highest concentration (30 μM) tested (190.9±53.8 pA v 160.6±35.3 pA; Fig 4A and 4B). Likewise, DCS had no effect on rise time (44.7±9.3 ms v 52.3±15.1 ms at 30 μM) of eNEPSCs. However, the drug elicited a clear, concentration-dependent decrease in decay time (Fig 4A and 4C). This was evident and significant even at low concentrations and the effect had an IC50 of 2.1 μM. The maximum normalised reduction (at 30 μM) was to 68.1±5.9% of control, corresponding to a decrease in mean decay time from 593.8±94.6 ms to 417.4±78.7 ms (*P*<0.01). The exemplar experiment shown in Fig 4A illustrates the lack of change in amplitude, but overlay of the records reveals the much faster decay in the presence of DCS.

**Fig 5:**
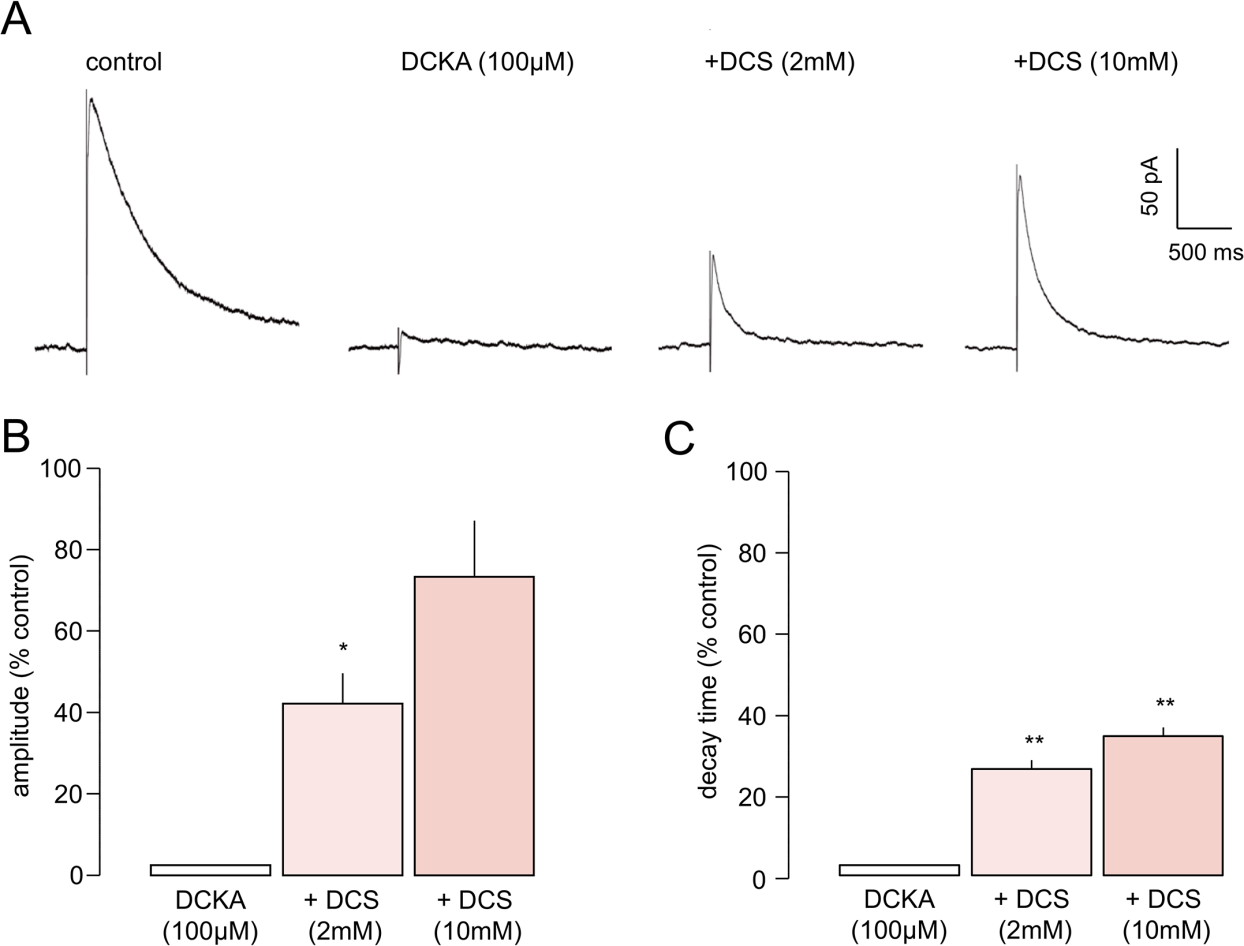
DCS rescues the abolition of the eNEPSC from inhibition by DCKA. **A.** Averaged synaptic responses from one neurone show abolition of the eNEPSC by the co-agonist site antagonist, DCKA. Subsequent addition of DCS at 2 mM partly restored the response; increasing the partial agonist concentration to 10 mM had a substantially greater restorative action. The histograms show the pooled normalised data for amplitude **(B)** and decay time **(C)** in 8 neurones. The restoration of the eNEPSC was partial, but it is noteworthy that the peak amplitude with DCKA plus DCS at 10 mM was not significantly different to that in control conditions. * *P*<0.05; ** *P*<0.01.

To confirm that DCS was exerting its effects at the co-agonist site of the NMDAr we determined the effect of combined administration of the co-agonist site antagonist, DCKA. We showed earlier that DCKA applied at 100μM abolished eNEPSC and this is illustrated again in Fig 5A. In the presence of the antagonist, subsequent addition of 1 mM DCS (n = 3) had no restorative effects on the eNEPSC (not shown). However, at higher concentrations, DCS rescued eNEPSC amplitude in a concentration dependent manner (Fig 5A and 5B; n = 8). As before, eNEPSCs were essentially abolished by DCKA, but in its continued presence the amplitude of the evoked response was increased by the partial agonist to reach a mean of 42.4±7.3% of control at 2 mM and 73.9±13.6% of control with cumulative addition to 10 mM (Fig 5A and 5B). The decay time of the restored responses was 26.1±2.9% of control (pre-DCKA) at 2 mM and 34.4±1.8% at 10 mM. Although the responses were not apparently fully recovered with 10 mM DCS, the eNEPSC at this concentration had a mean absolute amplitude of 188.4±101.9 pA, which was not significantly different to that before DCKA (217.6±64.5 pA). Thus, it seems likely that the effects of DCS alone on eNEPSCs were due to agonistic actions at the postNMDAr co-agonist binding site.

### The effects of DCS are reversed by D-serine

**Fig 6:**
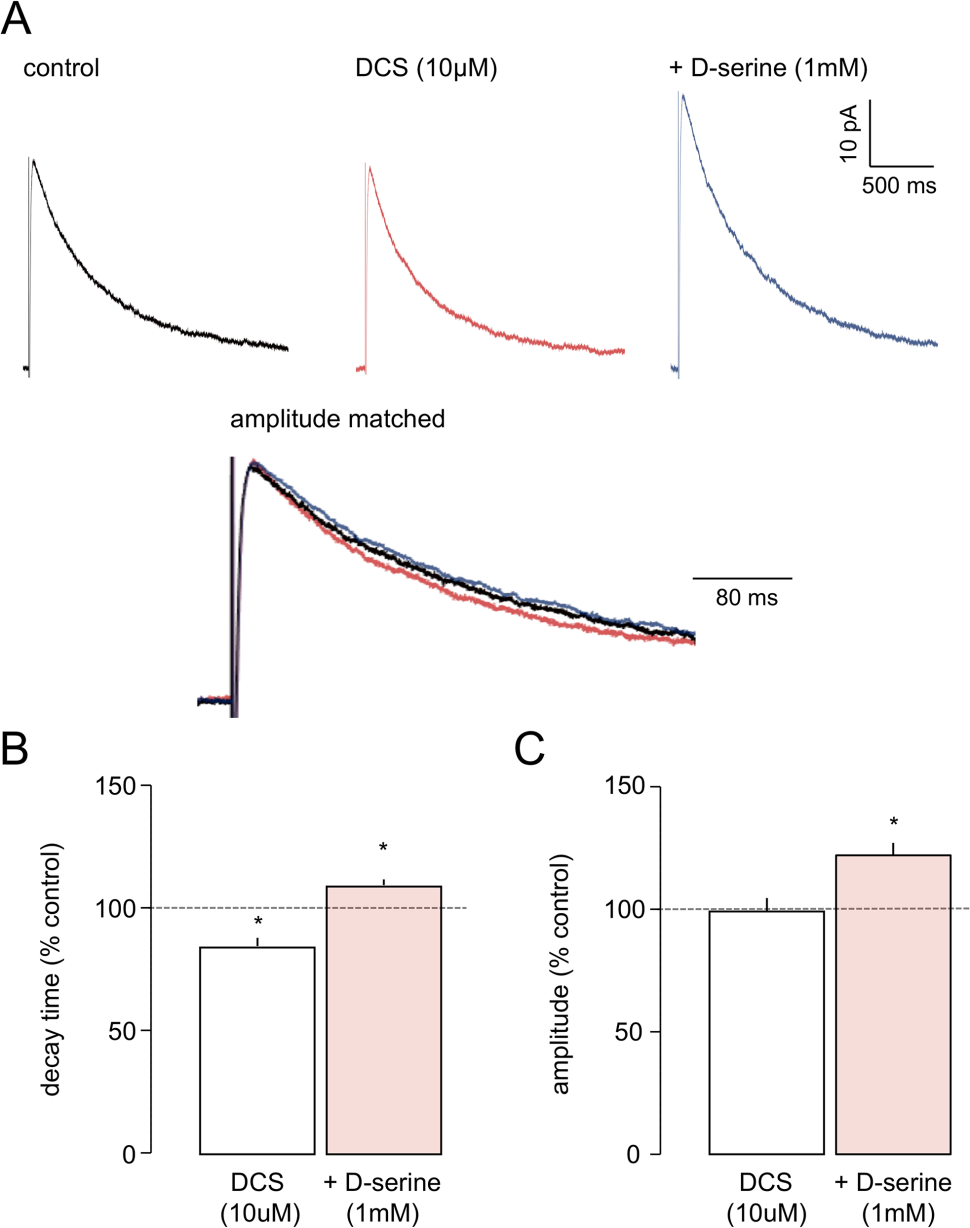
The effect of DCS on the eNEPSC is reversed by D-serine. **A.** Averaged responses in one neurone show little effect of DCS on amplitude but a decrease in decay time. Cumulative addition of D-serine reverses the effect of DCS and increases both parameters to beyond control levels. **B and C** show pooled data for the two parameters in 7 neurones. * *P*<0.05.

To confirm whether the effect of DCS on eNEPSC kinetics was due to its partial agonist action we examined whether it was reversible by the full agonist. In these experiments a sub-maximal concentration (10 μM, n = 7) of DCS was applied, followed cumulatively by a saturating concentration of D-serine (1 mM). As noted above, DCS elicited a selective and significant decrease in decay time (to 84.7±2.9% of control). Following the addition of D-serine (Fig 6A and 6B), the reduction of eNEPSC decay time by DCS was reversed and increased to beyond control levels (109.8±2.1%; *P*<0.05). In addition, as with D-serine alone (see above), eNEPSC peak amplitude also increased (to 126.6±4.0%) in the presence of DCS plus D-serine (Fig 6A and 6C). The mean values for eNEPSC in control, DCS (10 μM) alone and DCS (10 μM) plus D-serine (1 mM) conditions were 391.0±26.6 ms, 333.0±28.7 ms and 430.1±31.5 ms, respectively, for decay time, and 123.5±23.9 pA, 124.8±26.2 pA and 160.1±34.3 pA, respectively, for amplitude.

These results indicated that the actions of DCS were indeed readily reversible by a full-agonist and indicated that the effects of DCS are likely to be due to a partial agonism at the co-agonist site.

### Effects of ACBC at presynaptic NMDArs

**Fig 7:**
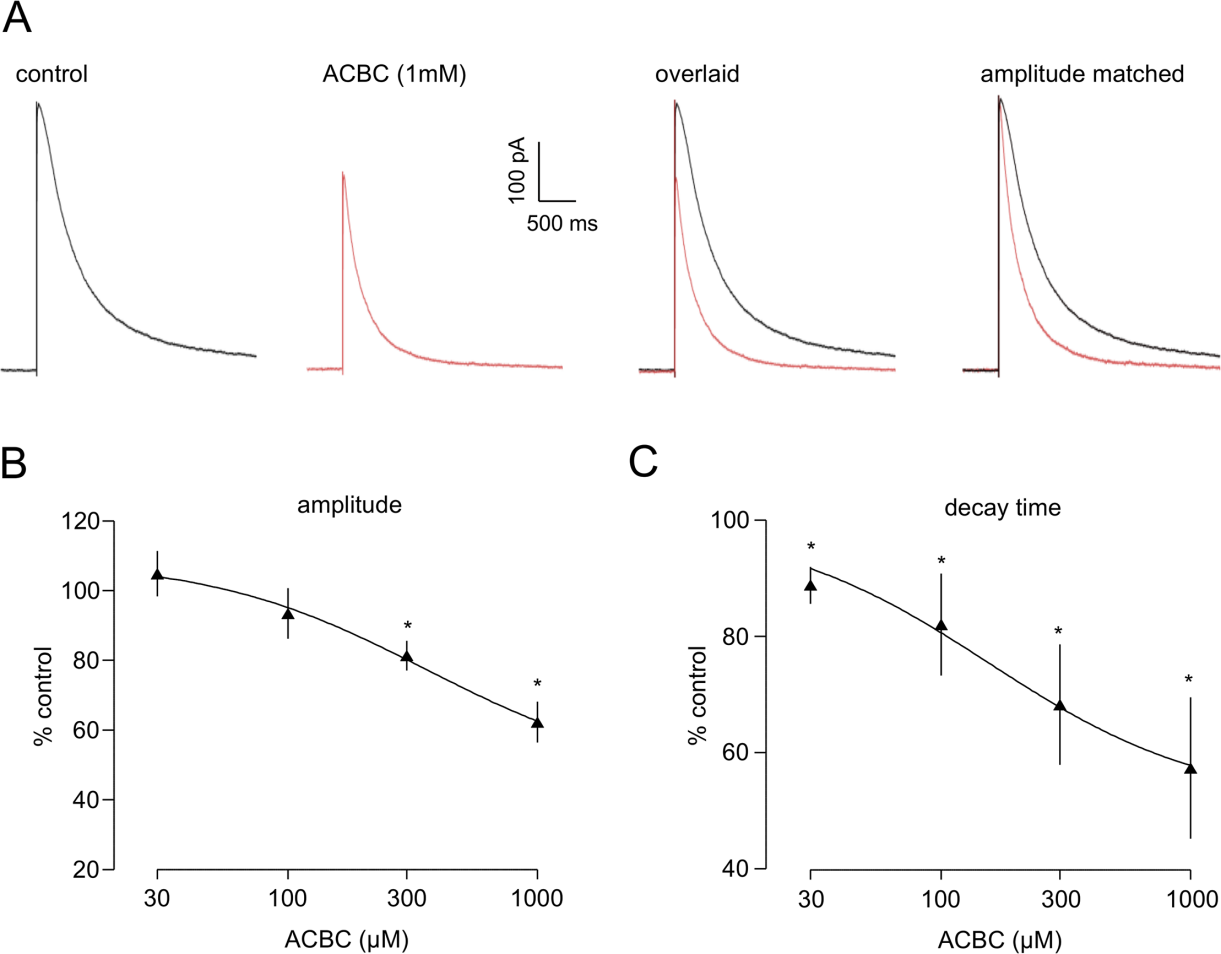
ACBC reduces preNMDAr activity. **A.** The histogram shows normalised frequency data for 5 neurones and indicates a significant decrease in frequency in the presence of the low-efficacy partial agonist. **B.** There was no concurrent change in frequency distribution of event amplitudes. **C.** The averaged mEPSCs recorded in one neurone illustrate that peak amplitude, rise and decay times of events was unaltered by ACBC. * *P*<0.05

DCS has a relatively high efficacy at the co-agonist binding site (~85%). To determine if efficacy was an important determinant of the actions of DCS we tested the effects of a partial agonist, ACBC, with a much lower efficacy (~40%; [43]) on both the preNMDAr and the postNMDAr (see below). ACBC (1 mM) decreased the frequency of mEPSCs to 69.4±3.9%, from 1.7±0.3 Hz in control conditions to 1.2±0.2 Hz in the presence of the drug (*P*<0.05; n = 5; Fig 7A). Concurrently, there was no change in frequency distribution of event amplitudes (Fig 7B) or in mean amplitude (6.9±0.3 pA v 6.5±0.3 pA; see Fig 7C). Likewise, neither mEPSC rise time (2.18±0.14 ms v 2.38±0.06 ms) nor decay time (2.85±0.26 ms v 3.27±0.23 ms) was altered by ACBC, exemplified by the averaged (n = 20) events illustrated from one cell in Fig 7C.

### Effects of ACBC at postsynaptic NMDArs

**Fig 8:**
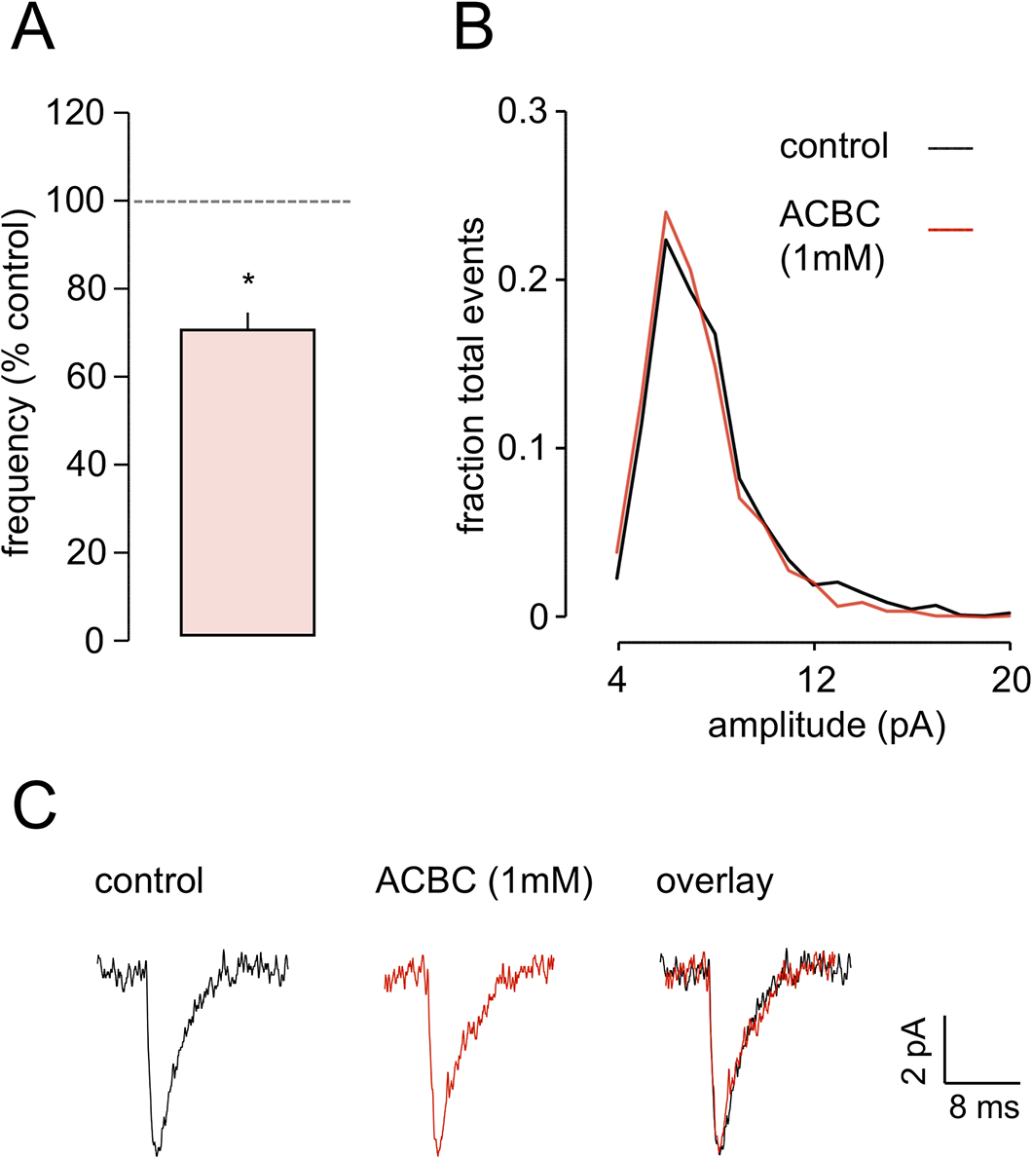
ACBC decreases both the amplitude and the decay time of the postsynaptic eNEPSC. **A.** Averaged responses for one neurone show a clear decrease in amplitude in the presence of ACBC, and the overlaid traces also indicate a substantial decrease in decay time. Amplitude scaling shows the latter effect very clearly. **B and C**. The graphs show pooled data for 7 neurones and illustrate the clear concentration-dependency for effects of ACBC on both parameters. * *P*<0.05.

Like DCS, ACBC (0.03–1 mM, n = 7) substantially reduced the decay time of eNEPSCs (Fig 8A and 8C). The effect of ACBC was approaching maximal at 1 mM, where we recorded a mean reduction in eNEPSC decay time to 57.5±12.4% of control, with a change in mean from 524.8±102.96 ms to 249.0±26.5 ms (*P*<0.05). In contrast to DCS, however, ACBC also reduced the amplitude of eNEPSCs in a concentration-dependent manner (Fig 8A and 8B). The mean change in peak amplitude at 1 mM was to 62.0±5.9% of control (239.4±47.3 pA v 141.5±21.2 pA; *P*<0.05). Rise time was unaltered (31.3±4.2 ms v 30.3±5.3 ms). The averaged responses shown for one neurone in Fig 8A clearly show the change in amplitude, and when the responses were amplitude scaled and superimposed the reduced decay time was also obvious.

## Discussion

DCS, the partial agonist at the NMDAr glycine/D-serine co-agonist binding site, had no detectable effect on the preNMDAr mediated tonic facilitation of glutamate release, whereas ACBC did induce a decrease in mEPSC frequency. This is explicable in terms of differences in efficacy of the two partial agonists. The preNMDAr is saturated, or very close to it, by endogenous D-serine [33], so the binding of a low efficacy partial agonist, ACBC, will substantially compete with the endogenous activation of the site. Since activation of the co-agonist site is obligatory for the receptor activation, ACBC will have a similar effect to a competitive antagonist at the site [33], and thereby reduce its activation by ambient glutamate. Thus, the tonic facilitation of glutamate release mediated by the preNMDArs will be reduced and mEPSC frequency will decline. DCS has a much higher efficacy (85%) than ACBC (40%), which means that its competition with endogenous D-serine will have a less deleterious effects on activation of the preNMDArs. Even complete blockade of preNMDAr activation with a competitive glutamate or co-agonist site antagonists only results in around 25–30% reduction in mEPSC frequency [30,31,33] it is not surprising that DCS did not elicit an obvious reduction in release.

It could also be that subunit specificity of the two partial agonists iis involved in the differential presynaptic effects of DCS and ACBC. There is evidence that although the action of partial agonists is due to binding at the co-agonist site on GluN1 subunits, efficacy may be determined by the identity of the GluN2 subunit. This certainly seems to be the case for DCS, which has a higher efficacy at GluN1-N2A (90%) than GluN1-N2B (65–70%) receptors [44]. PreNMDAr are comprised predominantly of GluN1-N2B diheteromers [31], whereas the postNMDAr are likely to be GluN1-N2A-N2B triheteromers [8]. Although the overall efficacy of ACBC is around 40% [43], comparative data at GluN1-N2A *v* GluN1-N2B receptors is not available. It is not inconceivable that it may have a much lower efficacy at the latter, and that this could contribute to why it is able to observably diminish the tonic facilitation of release whereas DCS does not.

In contrast to the preNMDAr, both partial agonists had significant effects on the response to postNMDAr activation. Following release, glutamate in the synaptic cleft rapidly peaks at approximately 1 mM, which is saturating at the postNMDAr, and is then cleared by reuptake with a time constant of 1 ms, approximately [45]. Activation of an NMDAr induces a series of openings that persist for a relatively long period due to a slow rate of glutamate unbinding. Glutamate rebinding does not occur [42,45] indicating that the time course of postNMDAr currents is determined only by receptor gating between bound/open, bound/closed and unbound/closed conformations [46]. Several gating mechanisms have been proposed for NMDAr, commonly with at least 10 transitions, though these are not resolvable from macroscopic currents [47].

Partial agonists at ligand-gated ion channels characteristically elicit submaximal synaptic currents even at full occupancy due to a decrease in receptor open probability that results from a reduction in the efficiency of receptor gating. However, partial agonists of NMDAr also alter the decay of NMDAr synaptic currents. These properties were extensively studied by Priestley and Kemp [48] who demonstrated that relative to a full agonist, partial agonists at either the glutamate or the co-agonist binding site decrease the decay time in an extent proportional to their efficacy. The kinetic analysis of Kussius and Popescu [49] has indicated that inter-subunit coupling is central to the NMDAr gating process and that partial agonism at either site occurs through broad effects on multiple gating transitions which results in receptors spending more time in bound/closed states. Therefore, the reason why eNEPSC peak amplitude and decay time are both determined by agonist efficacy is likely to simply be that both are determined by the efficiency of gating transitions. This model also accurately predicts that partial agonists accelerate the decay of macroscopic currents resulting from prolonged glutamate application by increasing the time spent in bound/closed states, which can also transition to long-lived bound/closed (desensitized) states. More recently this kinetic scheme was seen to accurately predict changes in the decay time of synaptic-like macroscopic currents resulting from structural changes in NMDAr induced by site-directed mutagenesis [50]. These studies suggest that the faster decay time induced by NMDAr partial agonists in comparison to full agonists could result from the same broad reductions in channel gating efficiency that underlie lower amplitude responses, providing a simple explanation of why both are determined by efficacy.

In the current study we found that partial agonists at the co-agonist binding site had different effects on parameters of eNEPSCs in native tissue that may be related to efficacy. DCS selectively decreased the eNEPSC decay time without affecting peak amplitude whereas ACBC decreased both decay time and peak amplitude. A parsimonious explanation for the lack of effect of DCS on amplitude is that the postsynaptic receptor co-agonist site is bound by the endogenous full agonist (probably D-serine; e.g. [51]), which has a higher efficacy than DCS, but is present at a concentration such that the number of co-agonist sites bound and, hence, endogenous activation of these sites is matched to the efficacy of DCS. Thus, when DCS is present, the number of glutamate receptors activated in response to stimulation is essentially unchanged but the macroscopic decay is altered.

In contrast, ACBC decreased both amplitude and decay time. This result would support the above scheme, since the low efficacy (~40%) would mean that there was a mismatch between efficacy and number of sites bound by the endogenous agonist, so that when the partial agonist is added it would be predicted to reduce amplitude as well as the decay of the macroscopic response. The increase in amplitude seen with exogenous D-serine, alone or in combination with DCS, indicates that the co-agonist sites at these synapses are probably not saturated. A saturating concentration of D-serine elicited an increase in amplitude of 15–25%, which suggests a tonic activation of around 80–85%. This agrees with the efficacy of DCS reported by Priestly and Kemp [48] who also studied eNEPSCs (although in rat neuronal cultures). Subsequently, Dravid et al. [52] have showed that inter-subunit coupling is a key determinant of the efficacy of DCS at different NMDAr subtypes observing that the efficacy of DCS is 90% and 65% of maximum for GluN2A and GluN2B containing NMDA receptors, respectively. Interestingly, we have previously demonstrated that the postNMDAr in the EC is likely to be a GluN1-N2A-N2B triheteromer [8]. In light of the findings of Dravid et al. [52] such a heterodimer could be anticipated to have the efficacy calculated by Priestly and Kemp [48].

Our data showing that D-serine increases the decay time of eNEPSCs warrants further discussion in light of evidence that D-serine is the endogenous co-agonist at synaptic NMDAr and, thus, already binding the receptor [51]. One explanation is the existence of partially-liganded NMDAr openings, which could cause a submaximal decay in the control condition but not when all sites are saturated by exogenous D-serine. Currently, partially-liganded NMDAr openings are not thought to occur, though evidence for this idea is largely reliant on expression systems, where regulation of the NMDA receptor may well be different. Our results with DCKA and DCS, where DCS rescued eNEPSC amplitude to a greater extent than decay time, may also support the existence of partially-liganded openings. A second explanation is that D-serine is not the sole endogenous ligand of the co-agonist site. Indeed, evidence for D-serine being the endogenous agonist at these receptors has been based on enzymatic depletion of D-serine, and typically this only produces a partial reduction of synaptic NMDAr currents [51]. Recently, endogenous activation of the postNMDAr coagonist site at hippocampal synapses has been shown to be due to a combination of both glycine and D-serine [53]; these may have different efficacies at the receptors in the EC, and this could account for a change in decay time when receptors become bound primarily by the exogenously applied D-serine. A further explanation is that D-serine is alters the contributions of different gating modes, which are argued to underlie the shape the synaptic response (see [54]). However, it would still be difficult to offer a molecular basis for this without either partially-liganded gated openings or the presence of another endogenous ligand.

An alternative explanation for the kinetic effects observed in this study is that the postNMDAr population present consists of two or more subtypes with varying decay time. A change in kinetics could then occur through biased agonist effects on these subtypes, altering the contributions of the subtypes to the overall response and, thus, the kinetics. Our results with DCS, where a kinetic change was seen independent of a change in amplitude, are not consistent with such a scheme and may argue against this explanation. However, for the results with ACBC and D-serine this remains a plausible basis for the observed kinetic effects. Previous studies have indicated that the receptors underlying these responses are predominantly a GluN1-N2A-N2B triheteromer [8], though without the use of knockout animals it is difficult to conclusively rule out the possibility of multiple contributing receptor subtypes.

In contrast to effects at postNMDAr the lack of effect of DCS on frequency of mEPSCs indicates that the partial agonist does not noticeably modulate preNMDAr function. The lack of presynaptic effect could be related to the difference in subunit composition of the pre and postNMDAr, as noted above. On the other hand, the preNMDAr appear to be saturated by endogenous D-serine [33], and even if this binding was completely replaced by DCS, we might expect only around a 20% reduction in function (see [52,54]), which may not be experimentally observable using the indirect reporting of receptor activity employed. This is supported by results with ACBC, which has a much lower efficacy and did decrease mEPSC frequency. Activation of preNMDArs does not appear to be restricted by saturation of the glutamate binding site, since blocking reuptake an increase the frequency of AMPAr mEPSCs [55]. Thus, extrapolating back from the postsynaptic site to presynaptic, it is possible that an unchanged strength of the current through the preNMDArs coupled with a moderately increased rate of closure in the presence of DCS would not affect physiological utility, as re-opening could quickly occur, and the tonic facilitatory effect would persist. With ACBC, both current strength and duration would be reduced and, thus, the facilitation would be compromised. Thus depending on the partial agonist, we may be able to differentially modulate the postsynaptic responses, but also exert site-specific (pre v post) modulation of NMDAr-mediated transmission.

In this study we have documented what is, superficially at least, a diminishing of postNMDAr-mediated transmission by partial agonists, be it a decrease in duration alone or in both amplitude and duration; in the case of ACBC, we also report a decline in preNMDAr facilitation of glutamate release. However, there is good evidence for cognitive enhancement with DCS [16–25] and other co-agonist site partial agonists (e.g. GLYX-13 [56]). Cognitive enhancement is generally targeted at elevating NMDAr function (e.g. [57]), so these results would seem counterintuitive. However, in preliminary studies (Lench A.M. and Jones R.S.G., see S1 Fig) we have examined the effect of DCS on synchronized local field potential discharges induced by block of GABA-inhibition in EC slices. The effects were complex but generally characterised by increased inter and intra burst frequencies and amplitude of discharges with low doses of DCS. We have not yet investigated the cellular basis of this in detail. However, since DCS only increases the rate of closure of postNMDAr, at synapses where NMDAr-mediated transmission is operative this could permit more rapid and frequent reopening concurrent with a maintained peak amplitude of the signal. Conceptually, this could result in enhanced synchrony. As synchronous network oscillatory activity is likely to underlie many forms of cognitive processing [58–60], this could be a basis for cognitive enhancement. We are currently investigating the cellular basis for the prosynchronous effects of DCS and other partial agonists.

In this regard, it is interesting that low doses of DCS have been suggested to be a useful adjunct or treatment to enhance cognitive processing in schizophrenics through enhancement of NMDA mediated transmission [61,62]. Based originally on the observations that ketamine and phencyclidine can induce a schizophrenia-like psychosis [63,64], the disorder is increasingly being seen as involving a hypofunction of glutamatergic, particularly NMDAr-mediated, transmission [65–67]. There is also increasing evidence that the cognitive disruption associated with schizophrenia may involve alterations in the oscillatory activity (particularly gamma-oscillations) in cortical networks (see [68]) and experimental studies have shown that NMDAr dependent gamma rhythms [69] may be substantially decreased in the entorhinal cortex in schizophrenia and related animal models [68,70]. Thus, it may be tempting to suggest that the counterintuitive prosynchronous effect of DCS that we propose here may be a basis for restoring normal oscillatory effects in the EC in schizophrenia and amelioration of cognitive deficits associated with it.

It is also interesting to note that the non-competitive NMDAr antagonist, memantine, mildly enhances cognitive processing in people with Alzheimer’s disease. This has been suggested to involve an increase in signal to noise ratio in cortical excitatory transmission signal, as a result of a reduction in NMDAr-mediated synaptic noise [71]. However, it is also the case that memantine can enhance both theta and gamma oscillations in cortical networks and reverse the deleterious effects on these rhythms induced by scopolamine [72]. We are not proposing that memantine and DCS share a common cellular mechanism for their prosynchronous effects, but it is clear that a negative effects on NMDAr mediated responses does not preclude a role as a cognitive enhancer, and the temporal and spatial location of these effects in network activity are likely to be determinant factors.

## Supporting Information

S1 Fig DCS enhances synchronised activity. Synchronised epileptiform bursts recorded extracellularly as local field potentials, were elicited in entorhinal slices by perfusion with bicuculline methiodide (20 μM), picrotoxin (50 μM) and strychnine (2 μM) to block both GABA_A_ and glycine receptors. Application of DCS at 30 μM, increased burst frequency and increased the duration of individual bursts as well as the frequency of discharges within the bursts. In some instances overall burst amplitude also increased. During washout, most parameters were rapidly reversed to control conditions although the intra-burst frequency increase was more persistent.
(TIF)
