# Supplementary figures and images for: Differential Effects of D-Cycloserine and ACBC at NMDA Receptors in the Rat Entorhinal Cortex Are Related to Efficacy at the Co-Agonist Binding Site
Source: PLoS One. 2015 Jul 20;10(7):e0133548. doi: 10.1371/journal.pone.0133548 (PMC4507855; doi:10.1371/journal.pone.0133548)

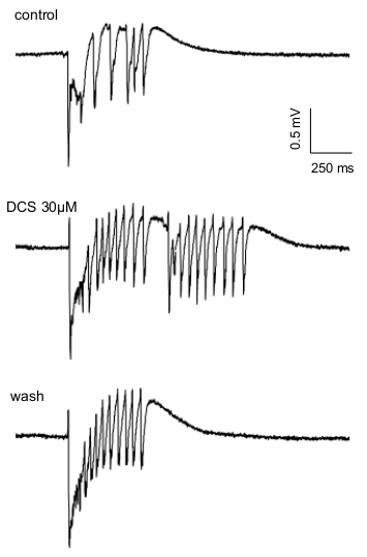

Supplement: S1 Fig — Synchronised epileptiform bursts recorded extracellularly as local field potentials, were elicited in entorhinal slices by perfusion with bicuculline methiodide (20 μM), picrotoxin (50 μM) and strychnine (2 μM) to block both GABAA and glycine receptors. Application of DCS at 30 μM, increased burst frequency and increased the duration of individual bursts as well as the frequency of discharges within the bursts. In some instances overall burst amplitude also increased. During washout, most parameters were rapidly reversed to control conditions although the intra-burst frequency increase was more persistent. (TIF) [file pone.0133548.s001.tif]
